# Now You See Me: Convolutional Neural Network Based Tracker for Dairy Cows

**DOI:** 10.3389/frobt.2018.00107

**Published:** 2018-09-19

**Authors:** Oleksiy Guzhva, Håkan Ardö, Mikael Nilsson, Anders Herlin, Linda Tufvesson

**Affiliations:** ^1^Department of Biosystems and Technology, Swedish University of Agricultural Sciences, Alnarp, Sweden; ^2^Centre for Mathematical Sciences, Lund University, Lund, Sweden

**Keywords:** animal tracking, computer vision, dairy cattle, precision livestock farming, convolutional neural network, automatic milking systems, animal identification, image analysis

## Abstract

To maintain dairy cattle health and welfare at commensurable levels, analysis of the behaviors occurring between cows should be performed. This type of behavioral analysis is highly dependent on reliable and robust tracking of individuals, for it to be viable and applicable on-site. In this article, we introduce a novel method for continuous tracking and data-marker based identification of individual cows based on convolutional neural networks (CNNs). The methodology for data acquisition and overall implementation of tracking/identification is described. The Region of Interest (ROI) for the recordings was limited to a waiting area with free entrances to four automatic milking stations and a total size of 6 × 18 meters. There were 252 Swedish Holstein cows during the time of study that had access to the waiting area at a conventional dairy barn with varying conditions and illumination. Three Axis M3006-V cameras placed in the ceiling at 3.6 meters height and providing top-down view were used for recordings. The total amount of video data collected was 4 months, containing 500 million frames. To evaluate the system two 1-h recordings were chosen. The exit time and gate-id found by the tracker for each cow were compared with the exit times produced by the gates. In total there were 26 tracks considered, and 23 were correctly tracked. Given those 26 starting points, the tracker was able to maintain the correct position in a total of 101.29 min or 225 s in average per starting point/individual cow. Experiments indicate that a cow could be tracked close to 4 min before failure cases emerge and that cows could be successfully tracked for over 20 min in mildly-crowded (< 10 cows) scenes. The proposed system is a crucial stepping stone toward a fully automated tool for continuous monitoring of cows and their interactions with other individuals and the farm-building environment.

## Introduction

According to latest reports (Rutten et al., 2013; Barkema et al., 2015), the average size of the dairy farm in Europe is continuously increasing which results in a more substantial number of animals for day-to-day control and caregiving. As daily farm work includes many different aspects, the time for observing the animals and finding those in need of additional care is dramatically decreased, which could lead to production diseases being unnoticed until later stages, requiring immediate veterinary attention (Geers and Madec, 2006; von Keyserlingk et al., 2009; Barkema et al., 2015). By assuring early detection of diseases and monitoring the health of the animals continuously and in real time, it is possible to increase the end value of the product for the consumer by creating animal-friendly production conditions. Studies are showing (Hermans et al., 2003; Herlin and Frank, 2007; Castro et al., 2012) that animals in pain or with the ongoing pathological conditions will express the deviations from their typical behaviors, which could be utilized as a valuable indicator for building the models describing animal's states of well-being. The sophisticated management practices and constant adjustments in the farm-building environment have also resulted in limited opportunities for dairy cows to express these natural/typical behaviors (Dominiak and Kristensen, 2017), obscuring the real clinical picture and welfare-related parameters.

To monitor farm animal's behavior and assess all the occurring interactions, one should be able to quantify and qualify performed interactions in a reliable, repeatable and continuous manner (Cangar et al., 2008; Porto et al., 2015; Guzhva et al., 2016). The focal observations and manual analysis of the recorded video material are two of the most common approaches used for these purposes. Such manual approach is time-demanding and is largely based on a skill of the person performing the annotations and interpretation of the performed behaviors. Another important feature is the ability to correctly identify the animals in overly crowded scenes, under varying illumination, during different hours of the day. The need for robust identification of individuals has become a multi-dimensional problem involving monitoring of production performance as well as individual health and the well-being of animals in dairy herds (Dziuk, 2003; Carné et al., 2009; Busse et al., 2015; Tullo et al., 2016). During the past decade, several alternatives for animal tracking and identification were proposed: WI-FI, RFID, GPS, ultra-wideband and Bluetooth-based products (Ahrendt et al., 2011; Nadimi et al., 2012; Rutten et al., 2013; Awad, 2016).

Among all methods mentioned above, RFID-modules gained considerable popularity over the course of past years due to certain advantages over the other methods. These advantages include the enormous potential for data storage, affordability and scalability, extended battery life. However, nevertheless all the advantages, RFID-modules do still require a considerable amount of work for setting them up: manual marking of animals with RFID-tags, protocols and infrastructure, integration into existing on-site digital ecosystem (Carné et al., 2009; Busse et al., 2015). Therefore, considering the increasing average size of dairy herds and number of individuals requiring monitoring, there is a need for a flexible and non-invasive system capable of alternative ways for individual tracking and identification (Banhazi and Tscharke, 2016).

As one of the alternatives, computer vision systems could ensure more frequent sampling, larger sequences recorded and analyzed (Cangar et al., 2008; Sellers and Hirasaki, 2014; Tullo et al., 2016). One of the other benefits of using computer vision system is the flexibility of the recording setup and a large number of features that could be extracted from the video material and used for descriptive analysis of the behaviors, locations of animals, identification and more (Guzhva et al., 2016). In a case of real-time monitoring and analysis, the need for extensive storage capacity is also resolved, as video stream could be assessed directly, making the procedure more efficient and suitable for practical on-farm use. With recent advances in the fields of computer vision and deep learning, as well as affordable computational power, systems based on computer vision could become the solution needed (Giot et al., 2013; Kulikov et al., 2014; Sellers and Hirasaki, 2014; Nilsson et al., 2015; Banhazi and Tscharke, 2016).

Most recent work on detecting cows have been focused on monitoring areas where the orientation of the cows was known due to physical properties of the surroundings. Two examples of this are the Viola-Jones based detector of Arcidiacono et al. (Porto et al., 2012) for detecting cows at the feed barrier and the work of Martinez-Ortiz et al. (Martinez-Ortiz et al., 2013) for detection and tracking of cow heads in narrow entrance corridors. Porto et al. (Porto et al., 2013, 2015) presented the current state of the art for detecting cows freely moving around. They also used a Viola-Jones based detector and needed six cameras at 4.6 meters height to cover a 15.4 × 3.8 m area to detect cows in three different orientations: vertical, horizontal and diagonal with a hit rate of 90%. General purpose object detection frameworks such as, YOLO (Redmon and Farhadi, 2017) and SSD (Liu et al., 2016) have outstanding performance. They do, however, focus on detecting objects of varying size and aspect ratio but with a fixed orientation (He and Lau, 2015; Ren et al., 2015). In the scenario considered in this paper, the size and aspect ratio are fixed and known, while orientation (rotation) of the object varies and have to be estimated.

This study aimed to create a flexible, state-of-the-art tracking algorithm for multiple objects. The near-real-time implementation in crowded scenes with varying illumination was considered one of the main priorities to ensure the viability in real-world scenarios.

## Materials and methods

### Study setup and recordings

All the video material for this study was recorded at a conventional dairy barn in the south of Sweden. The Region of Interest (ROI) for the recordings was limited to the waiting area with free entrances to four automatic milking stations (VMS, DeLaval) and a total size of the area−6 × 18 meters. There were 252 Swedish Holstein cows during the time of study that had access to the waiting area. With average (according to the statistics from VMS) milking rate of 2.4 per animal per day, the rough estimate for daily passage rate was 604.8 cows.

Video recordings were made using three Axis M3006-V cameras with a wide field of view, 134°. They were placed in the ceiling at the height of 3.6-meters, pointing straight down to optimize overview over the study area. Although the cameras were physically mounted to point fairly straight down, they were still slightly tilted. This tilting was synthetically removed during the rectification. The result of such calibration is video images where the cows have the same size regardless of where in the image they appear. Also, the scan-lines of the three different cameras become aligned, which allows them to be stitched together to form an overview of the entire waiting area.

**Figure 1:**
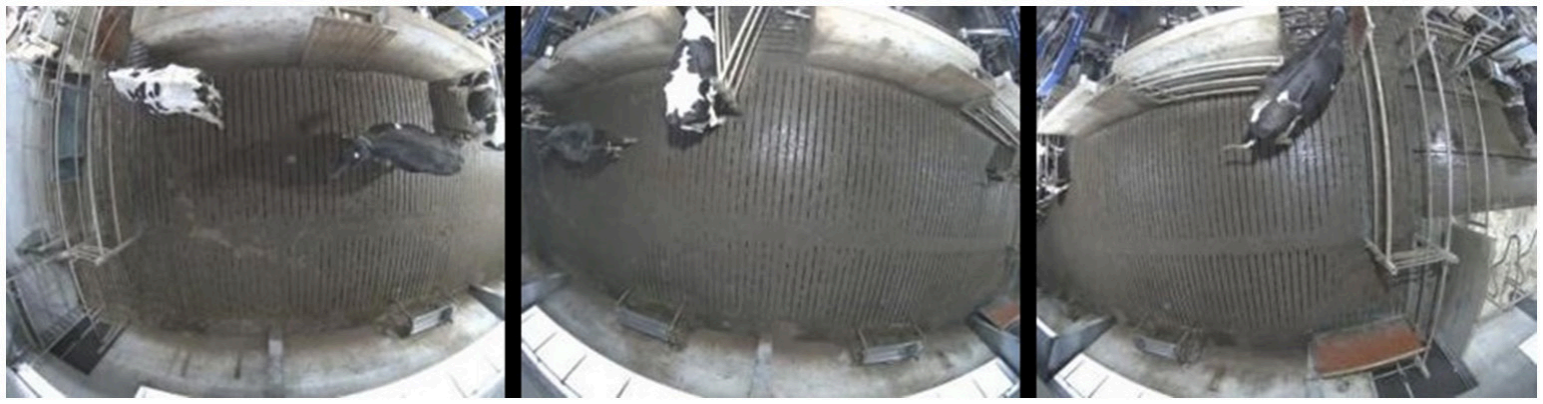
Example frames the recorded video.

The total amount of video data collected was 4 months, with a frame resolution 800 × 600 pixels, 16 Frames Per second (FPS) to provide quality similar to real-life situations where access to high storage capacities could be limited. These recordings contained 500 million frames collected continuously, 24 h per day and during two seasons (late autumn/winter for the first 3 months and spring for the last month), which gave the fair overview over different lightning/shadow conditions same as over different levels of activity during the day. Example frames from the setup are shown in Figure 1.

### Camera calibration

The classical pinhole camera model augmented with a lens distortion model was used to model the cameras (Hartley and Zisserman, 2004). The camera setup was calibrated by placing markers on the walls and stands in the middle of the waiting area. They were all placed at the same height and thus defined a plane. This is the plane in which all of the landmarks considered, except for the head, were expected to be found. By projecting detected landmarks back and forth between the camera images and this plane, detections from different cameras can be matched. The mean cow height in the barn was measured, and the plane was placed at the shoulder height. This height was estimated to be 1.49 meters with a standard deviation of 0.05 by measuring 12 random cows in the study area.

**Figure 2:**
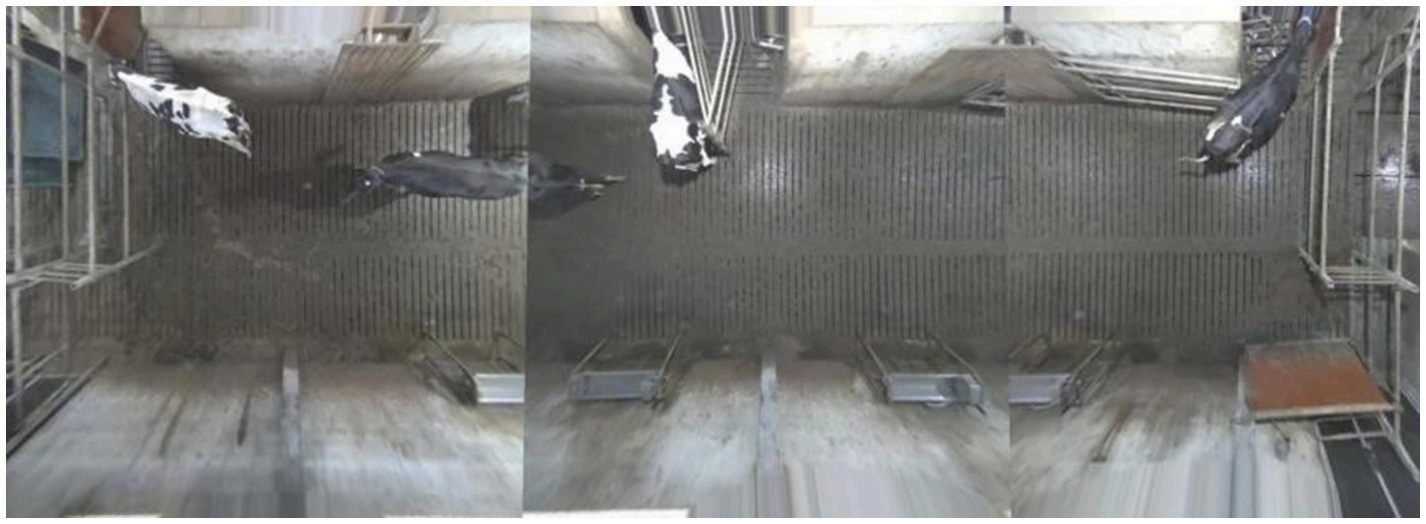
The frames from Figure 1 projected onto the cow shoulder plane and stitched together.

The lens distortion was removed, and a homograph that projected each of the camera images onto the cow shoulder plane was estimated. Figure 2 shows a view stitched together from all three camera images shown in Figure 1.

**Figure 3:**
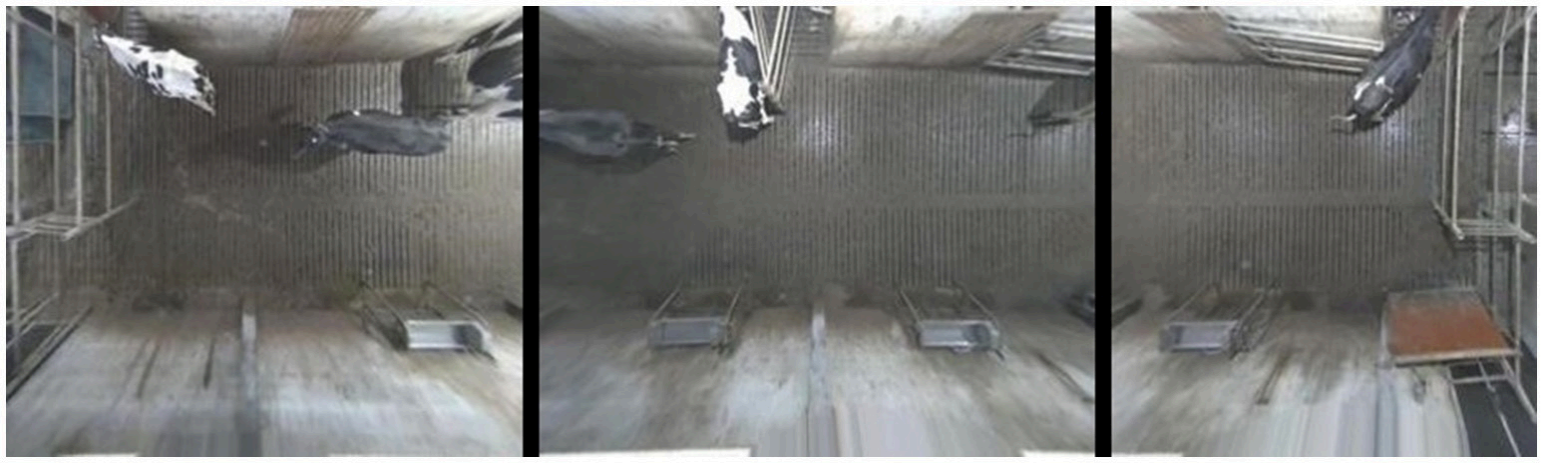
Dewarped frames from each of the cameras with overlaps to allow the detector to process them one by one.

This approach forms an overview of the entire waiting area. At the borders between the cameras, the image becomes strange, as cows positioned there are viewed from different directions on opposite sides of the border. However, this image is only used for illustrative purposes. There is enough overlap between the images to allow them to be processed one by one and then the resulting detections can be combined using the calibration. Figure 3 shows the separate dewarped frames used by the detector. Note how the same cow is almost entirely visible on both the left and the middle images.

### Training data for CNN-based cow-detector

The annotations from Ardo et al. (2018) were used. They consisted of 2,200 randomly sampled frames with every cow present being manually annotated. In total 9,279 cows were present and annotated. Each cow was annotated with seven landmark points correlated to anatomical points of interest (64,953 landmarks in total in this particular annotated subset; Guzhva et al., 2016). These landmarks represented following anatomical points of interest: cow head, left and right shoulder, front middle, left and right hip and back middle. There was one additional synthetic landmark “cow-center,” defined as a mean of front middle and back middle. This data was then used to train a CNN-detector (Ardo et al., 2018).

### The architecture of the CNN-based cow-detector

**Figure 4:**
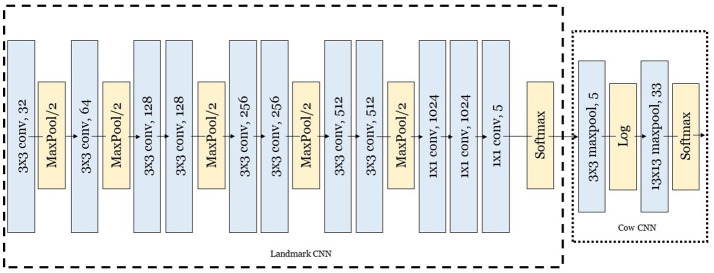
The architecture of the 2-step CNN cow detector. First CNN is used for landmark detection and the second one is used for actual cow detection (object and its orientation). The input for the first Landmark CNN is an image of any size [RGB channels scaled to the range (Rutten et al., 2013; Porto et al., 2015)]. The output of the Landmark CNN is a five-channel probability map which contains five different landmark classes: background, cow front middle, cow center, cow back middle and cow head. This five-channel probability map serves as the input for Cow CNN, resulting in another probability map as output. The output from the Cow CNN segments the original input image into either background or an object (cow) with known orientation.

One of the crucial prerequisites for robust tracking system is the detection of objects/cows. The CNN-detector used in this and one of our previous studies (Ardo et al., 2018) was implemented in two steps. The first step was a fully convolutional CNN that detects the landmarks in the image. The architecture of this network is a fully convolutional version of VGG (Simonyan and Zisserman, 2015). The second step was another CNN that works with the probability map produced by the first CNN as input to detect the cows and their orientations. The full circle is divided into 32 equally spaced orientations which generate 32 different oriented cow classes. These 32 different classes for orientation are needed to provide higher precision for additional “Behavioral Detector” module (Guzhva et al., 2016). Quite often, aggressive and positive interactions occurring between cows could only be separated by looking at exact location (and distance in between) of anatomical points involved in the interaction. Therefore, while distinguishing between different subtle behaviors, orientation class provides an additional level of interpretation of anatomical-point-alignment. In addition to that, there is the “no cow” class, which makes the total number of classes of this CNN equal to 33. The detector is fully convolutional, which means that it can be applied to images of any resolution, and the detector also is applied to all positions in the image in a sliding window fashion. The architecture of the detector is shown in Figure 4.

### State-of-the-art “tracker” algorithm for cows

#### The implementation of multi-object tracking

The Tracker optimizes over sequences of detection likelihoods produced by the CNN and is thus able to utilize all the information provided by the CNN, using per frame non-maximum suppression (NMS). The commonly used NMS technique is scenario-adjusted GreedyNMS, an algorithm where close-by detection-neighbors for specific objects are removed from probability map, leaving only detections with the highest score, to avoid multiple detections of the same object (Hosang et al., 2017). The tracking algorithm used the probability map produced by the CNN directly, without first constraining it to a few discrete detections. The probability map consists of probability, d_s,t_, of a cow being detected in each of discrete sets of possible states, *s* ∈ 𝕊, in frame t.

These states typically consist of the location of the objects (i.e., the coordinates of the probability map produced by the CNN), but could also be more informative as in the case above where the detector also detects the orientation of the cows. Each state, *s* ∈ 𝕊 then consists of a position (x; y) and an orientation α, i.e., s = (x; y; α) for some discrete sets of | 𝕊 | possible states.

The proposed tracking algorithm does not depend on the structure of those states and below 𝕊 refers to a general discrete set of states. The only assumption made about the states is that two different objects could not be in the same state at the same time, which makes sense as the position of the object typically is the part of its state.

The state space was augmented with a probabilistic motion model that described how the state of an object was allowed to move from one frame to another. This model was defined as a probability distribution, p(s_t_|s_t−1_), over states s_t_ in frame t given the state of the object, s_t−1_, in frame t – 1. Any such model could be used, but typically the model would assign high probabilities for the object to retain its current state or move to a neighboring state, while it assigns low probabilities to it jumping further away.

The gates described above were used to indicate when objects enter or leave the scene. Each gate was associated with a specific state. When an entrance gate, with state s_in_, indicated that a new cow had entered the scene, a new object was instantiated with state s_in_. Also, when an exit gate with state s_out_ indicated that a cow had left the scene, the object that currently is most likely to be in state s_out_ was removed from the scene. This means that the remaining parts of the tracker could operate under the assumption that the number of objects stayed known and constant from one frame to the next. For each state *s* ∈ 𝕊 the tracking algorithm could maintain o_s,t_, which is the identity of the object that is currently most likely to have the state s and p_s,t_, which is the probability that the object o_s,t_ has state s in frame t. These values were updated recursively by assuming that o_s, t−1_ and p_s, t−1_ are known and for each state s calculate the most likely previous state:

(1) $$e_{s}=\underset{\hat{s}}{\text{arg max }}p_{\hat{s},t-1}p(s|\hat{s})$$

This allows o_s, t−1_ to be propagated using:

(2) $$o_{s,t}=o_{e_{s}},t-1$$

To propagate the probabilities, the observation probabilities, d_s,t_, produced by the CNN detector are used:

(3) $${\tilde{p}}_{s,t}=d_{s,t}p_{e_{s},t-1}p(s|e_{s})$$

These propagated probabilities will no longer sum to one. By assuming that the object is still present and its state is one of the states for which it is currently the most likely object, a probability distribution for the current frame could be formed by normalizing the propagated probabilities:

(4) $$p_{s,t}=\frac{{\tilde{p}}_{s,t}}{\sum_{\hat{s}|o_{s,t}=o_{\hat{s},t}}{\tilde{p}}_{\hat{s},t}}$$

The second part of that assumption is an approximation. For distant objects it is insignificant, but for close objects, it might affect the results. Finally, the current state of each object, o, is estimated as:

(5) $$s_{o}=\underset{\hat{s}|o_{s,t=0}}{\text{arg max }p_{\hat{s},t}}$$

#### Real cow-ID from passive data-markers

The tracking algorithm presented in this study utilizes passive data-markers already integrated into modern dairy barn environment. Most of the manufacturers producing equipment for automatic milking systems have RFID-tags on animals, used for interactions with selection gates, milking stations, feeders. This means that the information required for identification of the individual cow is already present and saved in the computer logs every time animal moves/takes action in the barn. By combining these passive data-markers with a robust visual tracking system, non-invasive identification of individuals in different situations made possible.

As the real-ID of animals is usually controlled by the system of selection gates and there are usually several entries to the area of the interest, the opportunity to back-trace the real-ID number is higher and increases with every gate passage/equipment interaction per animal per scenario. The gates register when cows enter or exit the scene, and this information, together with the identification of the cow, is passed to the tracker.

However, for this study and to further investigate possible limitations of the proposed system, only one registration at automatic milking station was used for identification. The tracker detected and followed cows to the entrance to the milking station, where the system read the real-ID number. The detector then assigned this real-ID number to a detected cow and followed her along the tracklet backwards to the moment of actual entry to the waiting area.

## Results

### The performance of “tracker” algorithm

**Figure 5:**
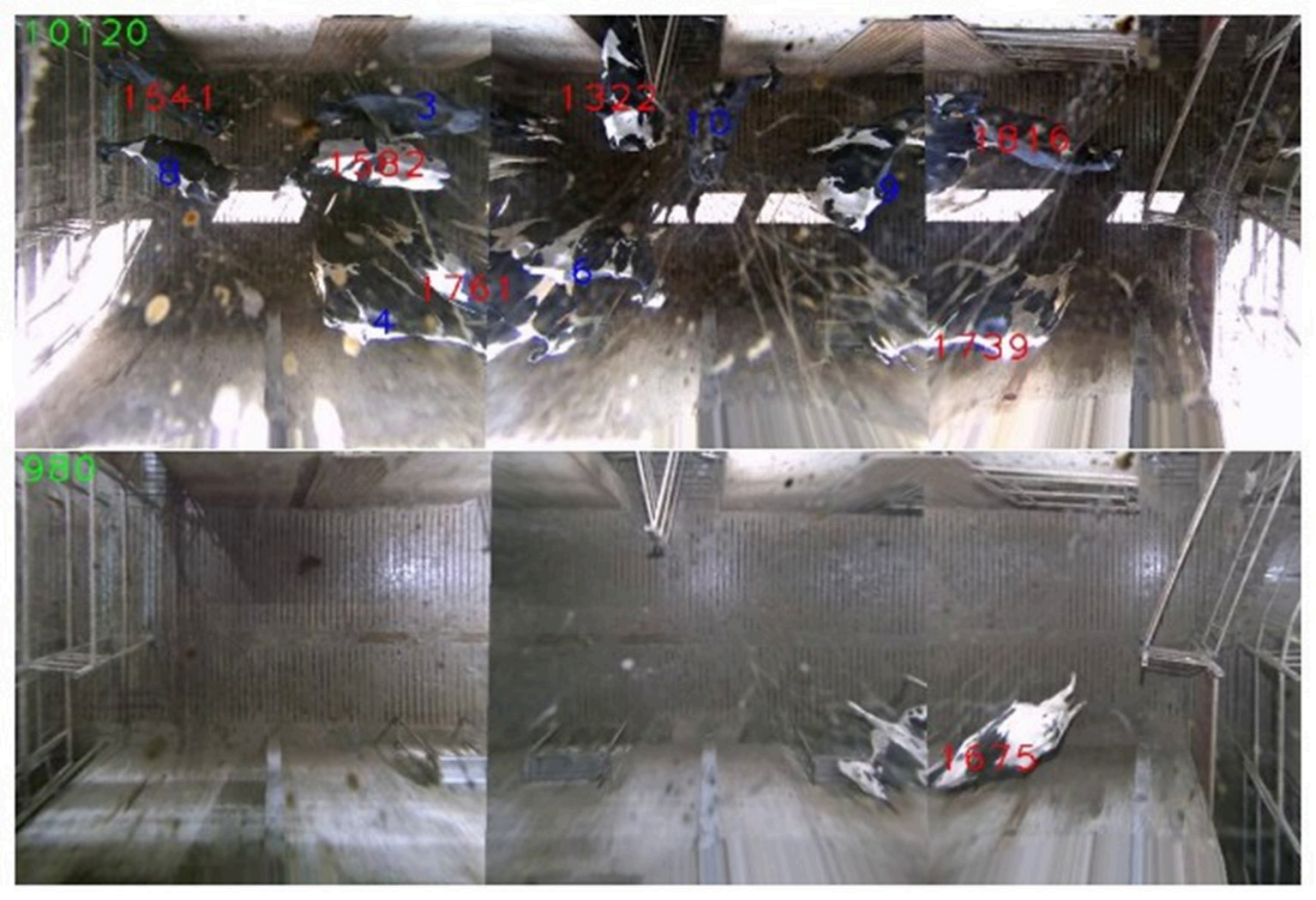
Example frames, with tracked objects marked, from the crowded, sunny (top) and easy (bottom) sequence. The red ID-numbers are initiated and assigned by the selection gates and placed on the correct cow by the tracker, while the blue numbers are placed manually in the first frame and then further tracked.

To evaluate the tracking system, two 1 h recordings were chosen. One recording with only a few cows in the waiting area during the night (with artificial lighting only) and another recording from a crowded scene (during the day when the sun shines in through the window, Figure 5). The exit time and gate found by the tracker for each cow, that both entered and exited the scene during the recording, were compared with the exit times produced by the selection gates.

**Figure 6:**
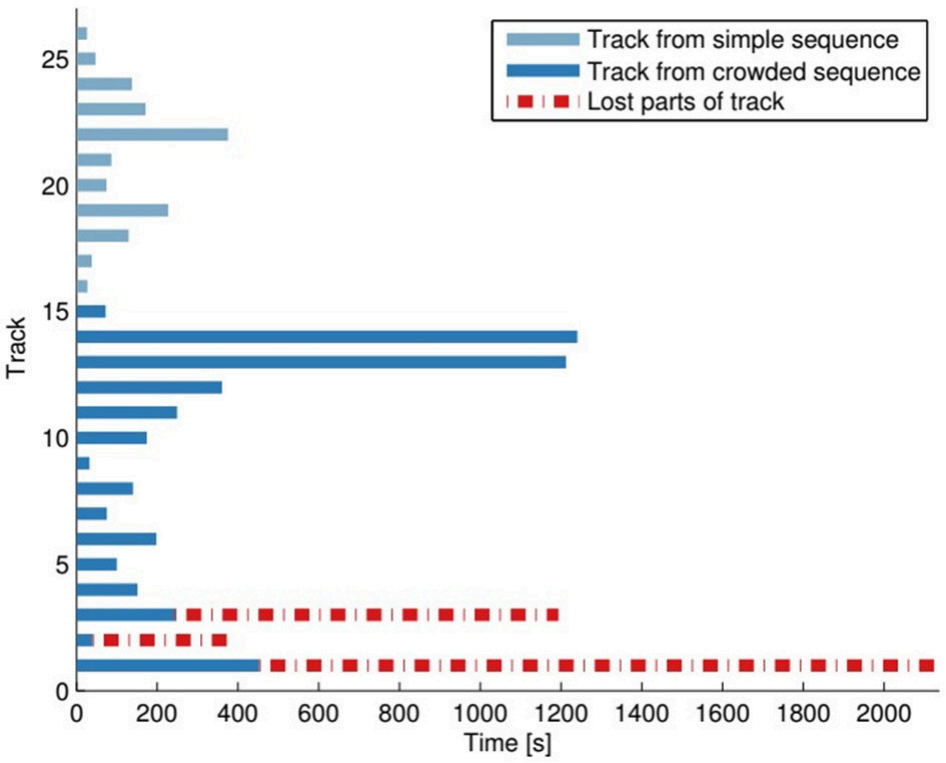
Here all 26 tracks in the dataset are shown in the *y*-axis and how long the tracker was able to follow each of them in the simple/crowded sequence.

This difference could be up to 60 s even for the correct tracks, as one of the gates was located outside the visible area. Results are shown in Tables 1, 2, respectively. A track was considered correct if cow left the scene through the “correct” gate and within 60 s of her RFID-tag registration by the respective gate. In total there were 26 tracks considered, and 23 were correctly tracked, while 3 of the tracks were lost at some point (no longer possible to confirm real-ID), cf. Figure 6. Note that some of these tracks were quite long and if a track is lost, it is highly unlikely that it will be found again. The longest successfully tracked sequence was 20 min long. The three tracks that failed were manually inspected to find the point in time where the error occurred. In one case tracker failed at the border of the image, at the overlap between stitched frames, most likely because cows were more distorted in this area from both viewing angles. Note also that two detections were merged in this overlapping area, after the camera calibration, which includes some errors. The other two cases were a case of ID-shifting due to a densely crowded scenario and confusion due to the earlier made error. Given those 26 starting points, the tracker was able to maintain the correct position in a total of 101.29 min or 225 s in average per starting point. Note that these numbers only show the complexity of the dataset. They should not be interpreted as a mean time to failure as most of the tracks are not lost entirely but detected at the exit borders of the scene.

**Table 1:** Complete trajectories of the simple sequence with columns indicating: cow id-number, tracker found the correct exit gate, time-difference between tracker exit and exit registered by the gate in seconds and the total length of the track in seconds.

**Cow-ID**	**Correct exit**	**Gate Diff. (s)**	**Track length (s)**
1832	1	2.38	26.06
1662	1	8.12	46.88
1733	1	6.44	137.44
328	1	3.88	170.81
1553	1	4.06	374.94
631	1	5.19	86.44
1761	1	42.88	73.94
1562	1	2.50	227.00
1852	1	56.12	129.19
1758	1	2.62	37.50
1803	1	22.94	27.06
1833	1	12.38	71.81

**Table 2:** Complete trajectories of the crowded sequence with columns indicating: cow id-number, tracker found the correct exit gate, time-difference between tracker exit and exit registered by the gate in seconds and the total length of the track in seconds.

**Cow-ID**	**Correct exit**	**Gate Diff. (s)**	**Track length (s)**
1582	1	1.62	1,240.44
1739	1	25.38	1,212.25
1390	1	5.19	360.31
1549	1	3.12	248.94
1767	1	0.75	173.94
1612	1	0.88	32.31
1776	1	1.31	139.56
324	1	3.12	75.00
1634	1	3.06	197.88
1527	1	1.25	99.94
1639	1	1.44	151.00
1792	1	764.75	1,193.56 (244.50)
1541	0	1,914.50	380.12 (40.00)
1761	0	60.62	2,126.75 (452.50)

*Three last rows are cases when the tracker fails. Within parenthesis is the length of the track in seconds that was successfully tracked before the tracker failed*.

## Discussion

The vast majority of current computer vision solutions for monitoring dairy cows are still in the developmental phase and do not provide the flexibility/functionality required for continuous monitoring of animals. The key-concepts forming the framework needed for robust solutions for automated and accurate tracking/identification of animals, as well as extended behavioral analysis features are also not fully established yet. Thus, investigating the opportunities and limitations of recent advances in computer vision and deep learning will facilitate the development of modules capable of monitoring animal health/welfare/behavior related parameters at low computational cost.

The “Tracker” module was developed and tested as the part of a pilot study, being a “proof-of-concept,” since the idea of using the passive data-markers for individual identification of animals was never tested before. Even considering the limited time available for the implementation of the “Tracker” module, a lot of potentially interesting information was gathered and separated into different classes for further development. The value of “non-invasive” continuous tracking system capable of identifying the individuals is tremendous and could help in resolving the common overstocking problems of modern dairy barns by assuring the optimal flow of animals and “benchmarking-on-the-go.”

In order to test the “Tracker” module, certain simplifications in the approach were taken. During the study, the exit gates did not register the exit event until the cow had been gone from the scene for a few seconds. Also, one of the entry gates was a place quite far outside of the observed area, which meant that the timings of the exit registrations were more reliable than the timings of the entry registrations. To mitigate the effect of this, the recorded video was reversed in time, and the exit gates were used as entry gates and vice versa. Also, synthetic observations with low probabilities were inserted at the entry and exit states when the actual detection there was lower than the synthetic one. These detections kept the cow tracks in those states during the time between the gate registration, and that enough of the cow appears in the image for a detection to be made. Also, the cows present in the scene at the start of the reversed video were manually marked and given a synthetic ID-number. This meant that no exit information was available for these cows. Instead, a different exit criterion was used (for all cows): if a cow's optimal position was one of the synthetic exit gate detections for more than 0.5 s consequentially, it was considered an exit and removed from the tracking. This means that the exit events from the gates were not used by the tracker and could instead be used to evaluate the results.

While considering the average duration of successful tracking events (approximately 225 s) and gradually decreasing accuracy in over-crowded scenes, one should bear in mind that the occurrence of errors (false-ID) do not indicate the limitations of the proposed solution. As mentioned previously, the identification error (when “Tracker” blends the real ID-numbers of cows that are in close proximity to each other) only indicates the “per-frame” failure. By extending the pool of potential data-collection points, one should be able to recover the initial detection and place the correct ID-marker on the object of interest. Our assumptions suggest that the system will benefit from more cameras installed all over the dairy barn, specifically around areas with selection gates or narrow passages, creating the extended “network” of passive data-markers.

Another potential add-on to the existing setup is to increase the resolution of recorded video material (step from default 800 × 600 pixels toward Full HD resolution) as that could increase the precision of detections and add new layers of information. However, with that in mind, the system should be still capable of recording the substantial amounts of data without increasing the storage cost. One possible solution for this could be to divide the range of features for monitoring into “immediate” (requiring lower resolution due to the simplicity of task) and “offline” (with higher resolution and additional information).

## Conclusions

▪ The study investigated and proposed the flexible and non-invasive computer vision system for tracking and identification of individual cows;
▪ The cows and their real-ID numbers were tracked in a waiting area before automatic milking stations;
▪ The system was deployed on a real conventional farm with all the real-world issues, such as, over year illumination changes and spider webs obscuring the field of view of the cameras;
▪ The proposed system is a crucial stepping stone toward a fully automated tool for continuous monitoring of cows and their interactions with other individuals and the farm-building environment;
▪ Furthermore, the system is based on several state-of-the-art deep learning methods, which enabled handling several real-world issues. Experiments indicate that a cow could be tracked close to 4 min before failure cases emerge and that cows could be successfully tracked for over 20 min.

## Ethics statement

Animals involved in this study did not interfere with the research equipment or research group, all the interactions with animals and/or their environment were approved by farmer and funding agency.

## Author contributions

OG, HA, and MN contributed conception and design of the study; OG did the data transfer, preparation and annotations necessary for the further analysis; HA and MN were responsible for algorithm implementation and evaluation; OG and HA were responsible for the evaluation of the results and system performance; OG wrote the initial draft of the manuscript and was responsible for further communication between authors as well as editing of the material. All the authors provided input for sections of the manuscript (concerning the field of expertise). All authors contributed to manuscript revision, read and approved the submitted version.

### Conflict of interest statement

HA was employed by company Axis Communications AB, Lund, Sweden. MN was part-time employed by company Axis Communications AB, Lund, Sweden. The remaining authors declare that the research was conducted in the absence of any commercial or financial relationships that could be construed as a potential conflict of interest.
